# Multiple HIV-1/M + HIV-1/O dual infections and new HIV-1/MO inter-group recombinant forms detected in Cameroon

**DOI:** 10.1186/s12977-016-0324-3

**Published:** 2017-01-13

**Authors:** Fabienne De Oliveira, Thomas Mourez, Aurélia Vessiere, Paul-Alain Ngoupo, Elodie Alessandri-Gradt, François Simon, Dominique Rousset, Jean-Christophe Plantier

**Affiliations:** 1 GRAM EA2656, Université de Rouen, Rouen, France; 2 Laboratoire de Virologie, Laboratoire associé au Centre National de Référence du VIH, CHU Charles Nicolle, 76031 Rouen Cedex, France; 3 Centre Pasteur du Cameroun, Yaoundé, Cameroon; 4 Infectious Diseases Data Observatory, Centre for Tropical Medicine and Global Health, University of Oxford, Oxford, UK; 5 Service de Microbiologie, APHP, CHU Saint Louis, INSERM U941, Faculté de Médecine Paris Diderot, Paris, France; 6 Laboratoire de Virologie, Institut Pasteur de le Guyane, Cayenne, Guyane française France

**Keywords:** HIV genetic diversity, M and O inter-group recombinant forms, Dual infections

## Abstract

**Background:**

Due to the prevalence of HIV-1 group M and the endemicity of HIV-1 group O infections in Cameroon, patients may be infected with both viruses and/or with HIV-1/MO recombinant forms. Such atypical infections may be deleterious in terms of diagnosis and therapeutic management due to the high divergence of HIV-1/O. The aim of this study was to identify prospectively such atypical infections in Cameroon.

**Results:**

Based on serological screening by *env*-V3 serotyping and a molecular strategy using group-specific (RT)-PCRs, we identified 10 Cameroonian patients harboring three different profiles of infection: (1) 4 HIV-1/M + O dual infections without evidence of recombinant; (2) 5 recombinants associated with one or both parental strains; and (3) 1 new recombinant form without parental strains.

**Conclusions:**

This work highlights the dynamic co-evolution of these two HIV groups in Cameroon that could lead to the emergence of a circulating recombinant form MO, and the need for accurate identification of such atypical infections for precise diagnosis, virological monitoring and therapeutic management with adapted tools.

## Background

The Human Immunodeficiency Virus type 1 (HIV-1) displays an extraordinary genetic diversity, divided into four groups (M to P) [1–4] due to simian origins, errors in reverse transcription, and a high recombinogenic potential. This latter accentuates diversity and evolution through the dynamic generation of multiple recombinant forms. At least 79 circulating recombinant forms (CRFs) and numerous unique recombinant forms (URFs) are now described for HIV-1 group M (HIV-1/M), accounting for almost 20% of all HIV infections (http://www.hiv.lanl.gov/, accessed in October 2016) [5]. Recombination is not restricted to HIV-1/M, since one intra-HIV-1 group O (HIV-1/O) recombinant as well as one HIV-2 CRF have also been reported [6, 7]. CRFs/URFs emerge in epidemiological conditions where two (or more) different strains predominate. The resulting dual (or multiple) infections [8] can generate recombinants that potentially spread in the population.

In Cameroon, besides the pandemic group M, all HIV-1 divergent groups described to date (N, O and P) are in circulation, with HIV-1/O representing about 1% of all HIV infections [9]. Despite the genetic distance between groups M and O, inter-group recombination is possible; three HIV-1/MO recombinant forms were thus reported in 1999 and 2004 in Cameroonian patients, also infected by the parental HIV-1/O and/or HIV-1/M strains [10–12]. The transmission potential of such recombinant forms was demonstrated by the description in 2010 of a fourth recombinant detected in the absence of parental strains in a Cameroonian patient living in France [13], and more recently by the transmission of a unique HIV-1/MO recombinant form in a Cameroonian couple [14].

These limited observations do not enable assessment of either the prevalence of these dual infections or the dynamic evolution and impact of their associated recombinant forms. But, it is known that presence of HIV-1/O in some part of the genome and/or in dual infections can have serious consequences at the individual level, raising future public health concerns. Indeed, HIV-1/O strains are characterized by high genetic diversity compared to HIV-1/M [6, 15] implying: (1) antigenic consequences that may result in false negative serological testing [16–18]; (2) genetic consequences that may lead to misquantification of the RNA viral load using group-M specific kits [19–21], and the need for specific assays for anti-retroviral (ARV) resistance genotyping [9, 22]; and (3) therapeutic consequences, with a natural resistance to Non-Nucleoside Reverse Transcriptase Inhibitors (NNRTIs) and poor predictive value of the genotypic resistance interpretation algorithms used for HIV-1/M [22–25].

Because HIV-1/O prevalence in Cameroon appears to have remained stable for many decades while HIV-1/M has expanded rapidly [26, 27], the co-circulation of these two different groups in the Cameroonian population may facilitate HIV-1/M + O dual infections and the genesis of HIV-1/MO recombinants.

The aim of this work was to identify prospectively such atypical infections in Cameroon, with an effective strategy combining a serological screening based on V3-serotyping and a molecular characterization using group-specific (RT)-PCRs.

## Methods

### Characteristics of samples

Between March 2006 and July 2009, 6796 serum samples were prospectively diagnosed HIV positive using a diagnosis strategy previously described as part of the routine laboratory activities of the Centre Pasteur du Cameroun (CPC) [28]. In the same period, 15,000 HIV plasma samples were received for viral load (VL) monitoring of previously diagnosed HIV positive patients. Buffy coats were also available for some patients.

### Serotyping screening of potential HIV-1/M + O dual infections

To discriminate between HIV-1/M or HIV-1/O mono-infections and HIV-1/M + O dual infections, all serum and plasma samples were screened with a serotyping ELISA test using a previously described method based on specific antigenic peptides of the *env* -V3 loop from both HIV-1/O (V3-O) and HIV-1/M (V3-M) [28].

### Molecular confirmation of HIV-1/M + O dual infections

**Fig. 1:**
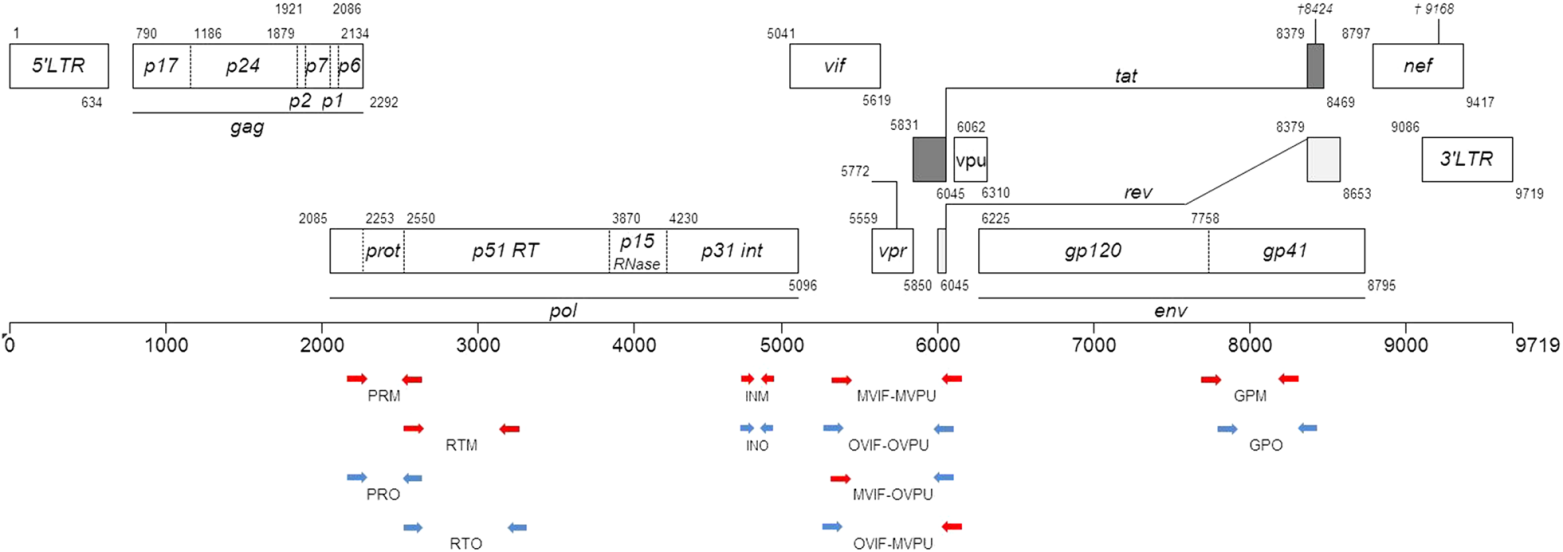
Position of HIV-1/O and HIV-1/M specific primers [cf materials and methods]. In *blue*, HIV-1/O target regions, in *red*, HIV-1/M. The lengths of the PCR fragment are respectively: PRM 507 pb, RTM 805 pb, PRO 452 pb, RTO 883 pb, INM 239 pb, INO 192 pb, MVIF-MVPU 891 pb, OVIF-OVPU 816 pb, MVIF-OVPU 815 pb, OVIF-MPVU 895 pb, GPM 656 pb, and GPO 674 pb. The position numbers are relative to HXB2 (GenBank accession number K03455)

All samples considered as dual seroreactive were further investigated using group-O and group-M specific PCRs or RT-PCRs, followed by sequencing. Due to the recombinant patterns previously published, four regions were targeted: the Protease (PR), Reverse Transcriptase (RT), and Integrase (IN) in *pol*; and the gp41 in *env*. The position of the different primers on the HIV-1 genome is summarized in Fig. 1. Primer sequences, amplified fragment lengths, and PCR conditions were previously detailed in former publications [6, 9, 22, 29–31].

### Molecular detection of HIV-1 M/O recombinant in *vpr*

As characterization of the partial or complete genomes of the three recombinant forms (described before the start of this work), revealed a breakpoint in *vpr* for two of them [10, 11], we hypothesized that this gene could be considered as a hotspot and it was included in our molecular strategy. Putative recombinants with a breakpoint in the Vpr region (designated as *vpr* recombinants) were investigated with a nested PCR protocol [10] that covered the region between the middle of *vif* to the middle of *vpu* (Fig. 1). Identification of *vpr* recombinants in both [M–O] and [O–M] patterns were made possible by the use of heterologous primers: MVIF/OVPU and OVIF/MVPU respectively. Presence of parental HIV-1/M and HIV-1/O strains was revealed by using the homologous primers MVIF/MVPU and OVIF/OVPU.

### Near full-length genome sequencing

Near full-length genome characterization was obtained from RNA extracted from two samples by the amplification of overlapping fragments using group-specific RT-PCR, followed by nested PCR and sequencing as previously described [14].

### Phylogenetic and recombination analyses

Group specific PCR fragments and *vpr* fragments were sequenced and aligned along with a set of different HIV-1/M and HIV-1/O reference sequences from the LANL database using MEGA 5.05 software [32]. *vpr* sequences from the recombinants described by Peeters et al. [10], Yamaguchi et al. [12], Vessière et al. [13] and Ngoupo et al. [14] were also included in the alignment (GenBank access No AJ239083, AY489738, GQ351296, KM438031 and KM438032). Genotyping was performed using HIV BLAST (http://lanl.hiv.org), Genotyping Retrovirus Tool (http://www.ncbi.nlm.nih.gov/retroviruses/) and REGA HIV-1 Subtyping Tool (http://dbpartners.stanford.edu/RegaSubtyping/). For recombination analyses, we performed: Similarity analysis, allowing localization of the recombination points, with SimPlot software [33]; and the Recombinant Identification Program (RIP) available on-line through the Los Alamos Database [34].

Construction of phylogenetic trees was performed using MEGA; genetic distances were calculated with the Kimura two-parameter method, and trees were obtained by the neighbor-joining method. The reliability of the branching order was estimated by 1000 bootstrap replicates.

## Results

### HIV-1/O mono-reactivities and HIV-1/M + O dual reactivities

During the 40-month period of the study, 61 of the 6796 HIV positive sera and 81 of the 15,000 plasma for viral load were reactive against at least the V3-O antigen. Among these 142 V3-O positive samples, 53 (37.6%) presented V3-O + V3-M dual seroreactivity.

### Molecular confirmation of HIV-1/M + O dual infections and detection of HIV-1/MO recombinant forms

HIV-1/M + O dual infection was defined as the presence of both HIV-1/O and M genomes in a sample; it was explored with group specific PCRs in 39/53 samples with dual seroreactivity. Fourteen samples were not tested because of a lack of material (plasma/serum or buffy coat). Molecular analysis showed that most dual reactivities with serological tests corresponded to non-specific cross-reactivities with V3-M or V3-O antigens. Among the 39 dual seroreactivities, 23 (59%) were positive only with group-O specific PCR in all of the four genes, and sequencing confirmed HIV-1/O fragments. These samples were considered as HIV-1/O mono-infections, i.e. presence of group O species only. Conversely, 6 (15%) were considered as HIV-1/M mono-infections, i.e. presence of group M species only.

**Fig. 2:**
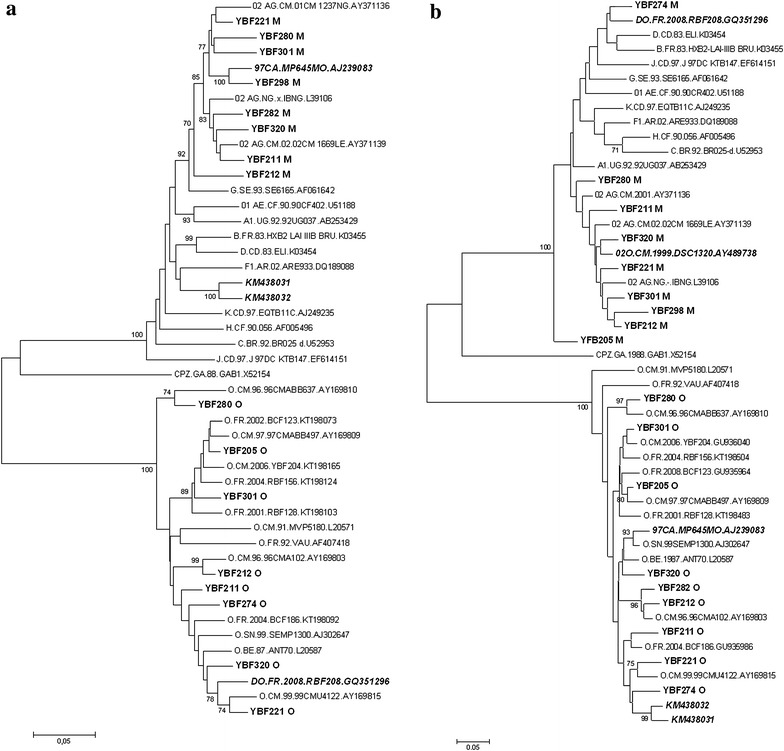
Phylogenetic trees of **a** the Protease-RT (899 bp) and **b** the gp41 (443 pb) regions. Sequences representative of HIV-1/M and HIV-1/O were downloaded from the Los Alamos database (hptt://www.hiv.lanl.gov). The four HIV-1/MO recombinants: (1) 97CA.MP645MO [10], (2) 02O.CM.1999.DSC1320 [12], (3) DO.FR.2008.RBF208 [13], and (4) KM438031 and KM438032 [14] are represented in *bold italics*. The new sequences obtained here are represented in *bold*. The gp41 HIV-1/O sequence of YBF298 could not be used for phylogenetic analysis, because of the relatively short length of the strain. SIVcpzGAB was used as the outgroup. Phylogenetic analyses were performed using MEGA software [32]: evolutionary distances were computed using the Kimura 2-parameter method; 1000 bootstrap replicates were performed to assess the reliability of the branching order. Bootstrap values are shown only when significant (>70)

Both HIV-1/M and O genomes were detected in 10 of the 39 (26%) patients. Molecular profiles and phylogenetic analyses are detailed in Table 1 and Fig. 2. *vpr* amplification and sequencing data allowed us to define three different profiles of infections:

**Table 1:** Molecular profiles of the 10 samples presenting a dual infection HIV-1/M+O and/or a HIV-1/MO recombinant form

Samples	Sample collection date	Protease	RT	Integrase	Envelope	HIV-1/O	HIV-1/M	*vpr*			
						Subgroup^a^	Subtype	HIV-1/O	HIV-1/M	HIV-1/MO	HIV-1/OM
YBF221	10/19/2006	M + O	M + O	M + O	M + O	H	CRF02	+	+	−	−
YBF280	NA	M + O	M + O	M + O	M + O	T	CRF02	+	+	−	−
YBF301^b^	10/16/2007	M + O	M + O	M + O	M + O	H	CRF02	+	+	−	−
YBF320^b^	NA	M + O	M + O	M + O	M + O	H	CRF02	+	+	−	−
YBF211^b^	05/04/2009	M + O	M + O	M + O	M + O	H	CRF02	+	+	−	+
YBF212^b^	12/11/2006	M + O	M + O	M + O	M + O	H	CRF02	+	+	−	+
YBF205^b^	09/13/2007	M + O	O	M + O	M + O	H	NT	+	−	−	+
YBF274	05/14/2008	O	O	O	M + O	H	D	+	−	−	+
YBF298^b^	11/06/2007	M	M	M	M + O	H	CRF02	−	+	+	−
YBF282	04/07/2007	M	M	M	O	H	CRF02	−	+	−	−

*NT* non typable, *NA* not available

^a^According to classification described by Leoz & al PLoS Pathog, 2015 (16)

^b^Amplification and sequencing of DNA from buffy coat

- dual infections without evidence of a *vpr* recombinant

Both HIV-1/M and O genomes were detected in four samples (YBF221, YBF280, YBF301, and YBF320). Both strains were characterized in the four genes using plasma (YBF221, YBF280) or buffy coat (YBF301, YBF320) (Table 1). The *vpr* PCR confirmed the results obtained with group specific PCRs, i.e. only the O–O and M–M homologous primers yielded an amplification fragment.

- *vpr* recombinants associated with one or both parental strains

**Fig. 3:**
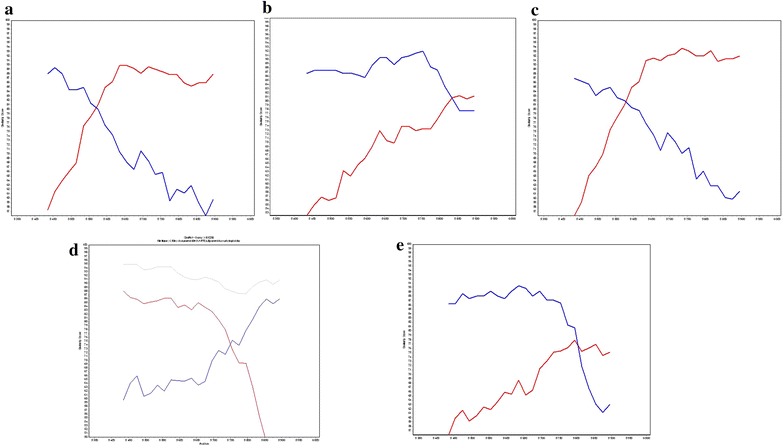
Similarity plots for *vpr* sequences of the 5 recombinant forms. Plots were generated by SimPlot software with 200 nucleotide (nt) windows, 20 nt increments, and the Kimura 2-parameter method with a transition-transversion (Ts/Tv) ratio of 2.0. Each of the 5 samples (YBF211 (**a**), YBF212 (**b**), YBF205 (**c**), YBF298 (**d**), and YBF274 (**e**)) was queried against an HIV-1/M subtype CRF02-AG (02_AG.IBNG.L39106) or subtype D (D.UG.92.92UG001.AJ320484) in *red*, and an HIV-1/O (O.BE.87.ANT70.L20587) in *blue*. YBF298 (**d**) was also queried against the recombinant form 97CA.MP645 (*grey line*) previously described by Peeters et al. [10]

We identified two samples (YBF211 and YBF212) for which both HIV-1/M and O strains were detected in the four genes. The sequence analysis of *vpr* fragments obtained with heterologous primers showed a recombination point in this gene (Fig. 3). For a third sample, YBF205, the results of the group-specific PCRs performed on the buffy coat indicated the presence of HIV-1/O sequences in all regions, and of HIV-1/M sequences only in the PR, IN and gp41 regions. The heterologous *vpr* PCR revealed the presence of an HIV-1 [OM] recombinant. The absence of amplification of HIV-1/M in two genes prevented us from concluding between a dual infection associated with a *vpr* recombinant or an HIV-1/O single infection associated with a recombinant with a complex pattern.

**Fig. 4:**
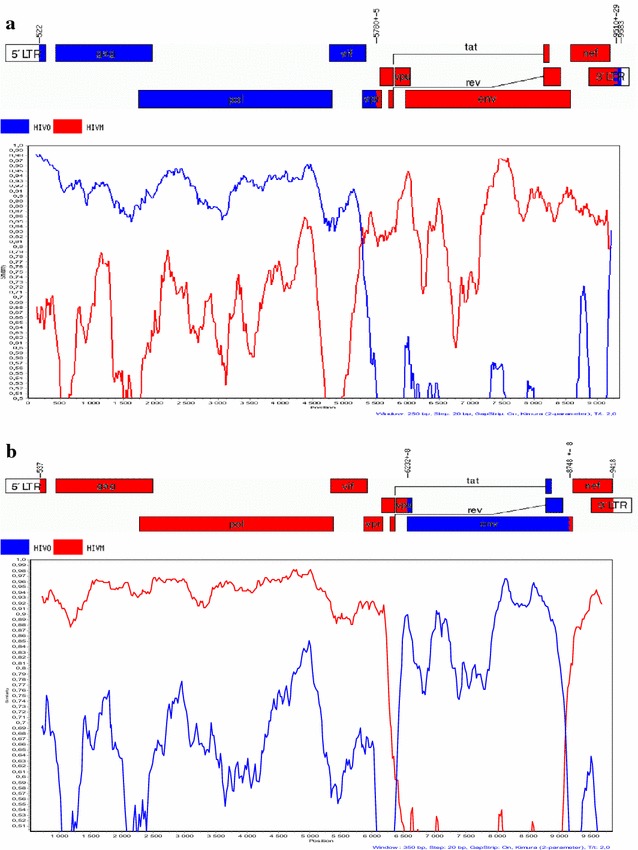
**a** Recombination pattern of the full-length genome of YBF274. Analysis was performed using SimPlot software with 250 nucleotide (nt) windows, 20 nt increments, and the Kimura 2-parameter method with a transition-transversion (Ts/Tv) ratio of 2.0. Sequences representative of a strain of HIV-1/M (D.CD.1983.ELI_patent.A07108 in *red*) and O (O.CM.98.98CMA104.AY169802 in *blue*) were used. The genome map was obtained using the Recombinant HIV Drawing Tool (http://www.hiv.lanl.gov/content/sequence/DRAW_CRF/recom_mapper.html). **b** Recombination pattern of the full-length genome of YBF282. Analysis was performed using SimPlot software with 350 nucleotide (nt) windows, 20 nt increments, and the Kimura 2-parameter method with a transition-transversion (Ts/Tv) ratio of 2.0. Sequences representative of a strain of HIV-1/M (02_AG.NG.IBNG-patent DD409979 in *red*) and O (O.CM.98.98CMA104.AY169802 in *blue*) were used. The genome map was obtained using the Recombinant HIV Drawing Tool (http://www.hiv.lanl.gov/content/sequence/DRAW_CRF/recom_mapper.html)

For two other patients (YBF274 and YBF298), the results of the group specific PCRs or RT-PCRs of the *pol* and *env* genes were discordant, with only one strain amplified in *pol* (HIV-1/O strain for the YBF274 sample and HIV-1/M strain for YBF298), and the two populations, M and O, in *env*. These profiles associated with the results of *vpr* amplification indicated a *vpr* recombinant, associated with only one parental virus (Table 1). For YBF274, sequencing of the complete genome of the recombinant form, showed a mosaic pattern with *gag*-*pol* belonging to group O (subgroup H) and *env* belonging to group M (subtype D), with two breakpoints, in *vpr* and in LTR (Fig. 4a). For YBF298, similarity analyses showed homology with the recombinant form 97CA.MP645 previously described by Peeters et al. [10]. Indeed, an HIV-1/M fragment was detected in the four regions as well as a group O fragment in the envelope (Table 1). The sequence alignment and the recombinant [MO] profile in the *vpr* gene showed a recombination breakpoint at the same localization as for 97CA.MP645 (Figs. 2 and 4). This lead us to conclude to a single HIV-1/M infection associated with the presence of an [MO] recombinant pattern (Table 1), as for 97CA.MP645. Phylogenetic analysis showed a strong link between YBF298 and 97CA.MP645 in the HIV-1/M *pol* fragment (Fig. 2).

- Recombinant form only with breakpoint outside *vpr*

For the YBF282 sample, group specific PCRs were discordant in *pol* and *env*, suggesting the presence of a [MO] recombinant virus (Table 1). However, only the presence of an HIV-1/M fragment was detected in the targeted *vpr* gene. These results suggest the existence of a recombinant virus with the breakpoint situated downstream of *vpr*. Sequencing of the complete genome confirmed the pattern *pol* M (subtype CRF02_AG) and *env* O (subgroup H), with two breakpoints, in *vpu* and at the end of gp41 (Fig. 4b).

### Characterization of the ARV resistance profiles of the Protease and Reverse Transcriptase regions of the HIV-1/O and HIV-1/M fragments

Partial amplification of *pol* gave us the opportunity to analyze the ARV resistance profiles of these new strains involved in multiple infections associated or not with recombinant viruses. We performed interpretation of resistance of the HIV-1/M and HIV-1/O species according to v25 of the ANRS algorithm for interpretation of resistance (http://www.hivfrenchresistance.org/). The HIV-1/M PR sequences harbored mutations conferring resistance or possible resistance to nelfinavir and/or saquinavir, for three of the samples (YBF301, YBF205, and YBF298) (Table 2). On the RT sequences, resistance mutations were observed only for YBF212, leading to resistance to rilpivirine and possible resistance to etravirine. Many more mutations for PR and RT of HIV-1/O species were observed, leading to: (1) resistance or possible resistance to atazanavir, indinavir, lopinavir and saquinavir; (2) resistance to all NNRTI classes for 6 of 8 viruses (YBF221, YBF301, YBF320, YBF211, YBF212, YBF274) due to the presence of Y181C residue; and (3) resistance to lamivudine/emtricitabine and possible resistance to abacavir for two of the samples (YBF221 and YBF301) due to the presence of M184V residue.

**Table 2:** Resistance profiles of the 10 samples presenting a dual infection HIV-1/M+O and/or a HIV-1/MO recombinant form

Samples	Profile	Protease	RT	Resistance associated mutations and resistance interpretation^a^					
				HIV-1/M species			HIV-1/O species		
				Protease	RT	Resistance profile^b^	Protease	RT	Resistance profile
YBF221	Dual	M + O	M + O	K20I M36I L63P/L L89M	V90I		L10V L33I M36I M46L/M I62V A71V K20C L63T L89T	T69S M184V A98G V179E Y181C L210Y	ATV, IDV, 3TC/ FTC, NNRTI *LPV, SQV, ABC*
YBF280	Dual	M + O	M + O	K20I M36I V77I L89M	None		L10V V11I I15V M36I I62V A71V K20C D60N/S L63I L89I	A98G K103R V179E L210Y	SQV *ATV*
YBF301	Dual	M + O	M + O	L10V K20I L24L/I M36I L89M	None	SQV	L10V I15V M36I I62V A71V K20C L63T/A L89M	M184V A98G K103R V179E Y181C L210Y	SQV, 3TC/FTC, NNRTI *ATV, ABC*
YBF320	Dual	M + O	M + O	K20I M36I L89M	K101Q		L10I/V I15V M36I I62V A71V K20C L63T L89I	A98G V179E Y181C L210Y	SQV, NNRTI *ATV*
YBF211	Dual + recombinant	M + O	M + O	I15L K20I M36I L63P L89M	None		L10V I15V M36I I62V A71V K20C L63T L89I	A98G V179E Y181C L210Y	SQV, NNRTI *ATV*
YBF212	Dual + recombinant	M + O	M + O	K20I M36I L89M	E138A V179I K101Q	RPV *ETR*	L10I I15V L33V M36I I62V A71V/I K20C F53Y/C D60S L63A L89I	A98G V179E Y181C L210Y	ATV, SQV, NNRTI
YBF205	Dual + recombinant	M + O	O	K20I L24L/I M36I L89M		*SQV*	L10I I15V M36I I62V A71V K20C D60N/S L63T L89I	A98G K103R V179E L210Y	SQV *ATV*
YBF274	Single + Recombinant	O	O	–	–		L10V/I I15V M36I D60E I62V A71V K20C L63T L89I	A98G V179E Y181C L210Y	ATV, SQV, NNRTI
YBF298	Single + recombinant	M	M	K20I D30N M36I D60N G73S L89M	None	NFV *SQV*	–	–	–
YBF282	Recombinant alone	M	M	G16E K20I M36I L63P/L L89M	None		-	-	

^a^According to the ANRS resistance interpretation’s algorithm v25

^b^In underline = resistance; italic = possible resistance

## Discussion

The aim of this work was to detect HIV-1/M + O dual infections and inter-group recombinant forms in Cameroon. We designed a sero-molecular strategy based on three hypotheses: (1) M + O dual infections are the pre-requisite for the genesis of HIV-1/MO recombinant forms (as the three recombinants described by 2004 were in patients with parental strains [10–12]; detecting dual infections may facilitate detection of recombinants), (2) *vpr* is a hotspot of recombination between HIV-1/M and HIV-1/O (two of the three MO recombinants forms exhibited a *vpr* breakpoint [10–12], and (3) since a breakpoint was found two times in *vpr* and once in the Integrase, the mosaic pattern could be as simple as *pol* O–*env* M or *pol* M–*env* O, so that performing molecular analyses in these regions could be sufficient to detect putative recombinant forms.

The first step was to perform serotyping screening based on V3-O and V3-M antigens, to discriminate between HIV-1/M or HIV-1/O mono- and dual- seroreactivities easily and rapidly among the 21 796 samples available. We detected 142 samples with reactivity at least against the group O antigen, among which 53 (37.3%) presented with M + O dual reactivities. As previously described, this serotyping can present cross-reactivity [35], since 29/39 samples were concluded as mono-infection by PCR (HIV-1/M: n = 6; HIV-1/O: n = 23). Therefore, dual seroreactivity must not be systematically concluded as dual infection. Despite these limitations, our results showed that our serotyping screening strategy was useful for selecting samples with possible dual infections with or without recombinant forms for further additional molecular exploration.

The second step was to perform molecular investigation using group-specific PCRs. Among the 10 samples with dual seroreactivities, we identified: (1) 4 HIV-1/M + O dual infections without evidence of a *vpr* recombinant; (2) 5 *vpr* recombinants associated with one or both parental strains; and (3) 1 *vpu* recombinant without parental strains. All these cases are new except for YBF298, which could correspond to the 97CA.MP645 strain previously described or to this strain transmitted to another patient with an epidemiological link.

The diversity patterns of the six recombinant forms were coherent with HIV molecular epidemiology in Cameroon, since they implied parental HIV-1/O subgroup H (6/6) and HIV-1/M subtype CRF02_AG (4/6, 67%), the most prevalent forms of each group [15, 36]. Thus, our work has shown that the co-circulation of HIV-1/M and HIV-1/O leads to dual infections and recombinant forms that could correspond to as many URFs_MO as described for HIV-1/M. These data, and our recent report of the transmission of an URF_MO in a couple, highlight the fact that recombinants are circulating in Cameroon, are more numerous than initially thought, and that a CRFs_MO or _OM could emerge in the next years. This hypothesis could be supported by the previous report that an MO recombinant form is more replicative than the parental strains [10].

The impact on public health of these dual infections and recombinants is difficult to predict. Paradoxically, even though we found a relatively small number of dual seroreactivities (53 among the 21 796 samples), these atypical infections accounted for 26% of dual seroreactive samples, highlighting that these phenomena are not as rare as previously thought. The negative impact of the genetic diversity of group O on serological diagnosis, resistance to NNRTI-based treatments and RNA plasmatic misquantification is now well-known. Similarly, the circulation of M + O dual infections and/or mosaic strains including group O fragments could have deleterious consequences. Thus, recombinant forms with a group O envelope (in absence of parental forms) will not be diagnosed by serological tests that are not adapted to this group due to antigenic variability [17, 18, 37], thus enabling silent spreading of these viruses. Moreover, if M + O dual infections and recombinants with group O fragments (particularly in RT) are unknown, unadapted therapeutic management could lead to rapid virological failure of the cART due to the natural resistance of group O to NNRTIs, and subsequently to the emergence of strains resistant to other partner drugs. We have shown here that some HIV-1/M species and many HIV-1/O species are resistant or possibly resistant to many drugs; some of these mutations are classically found in HIV-1/M non-B subtypes or correspond to the natural polymorphism of HIV-1/O [22]. But, some others are selected under pressure of cART, as M184V or D30N for example, indicating that even in absence of therapeutic data, patients may have had a cART. This natural polymorphism together with selected mutations as found in YBF221 and YBF301 dual infections (Table 2) is of concern if a recombinant form emerges with the virological properties of HIV-1/M associated with the multi-resistance pattern of the HIV-1/O species. Another concern is the virological follow-up, which if performed with a group specific technique, may underestimate the viral populations according to the amplified regions; paradoxically, the development of non-specific viral load kits (RealTi*m*e HIV-1, Abbott; Cobas TaqMan HIV-1 v.2, Roche for example) will quantify global M + O populations without distinguishing them, and will lead to the non-identification of recombinants. As a consequence, knowledge of these particular cases of multiple and recombinant infections is essential for accurate virological monitoring and therapeutic management with specific tools. We already observed an HIV-1 group M superinfection in an HIV-1 group O-infected patient in a pregnancy context [38].

At an individual level, these dual infections and recombinant forms also raise questions about their natural evolution, especially concerning pathogenesis compared to HIV-1/M or HIV-1/O mono-infections.

Our data reveal that the molecular epidemiology of HIV in Cameroon is becoming more complex; this is not surprising due to the wide genetic diversity of viruses circulating in this region, but the situation is maybe even more complex. Indeed, our work has several limitations that could underestimate the prevalence of M + O dual infections and recombinant forms. The sample collection, performed from 2006 to 2009 and not according to epidemiological methodology, may not be representative of the current situation and cannot define the prevalence or frequency of such forms. We only analyzed M + O dual seroreactivity, but reports of two cases with recombinants only [13, 14] and a third case here (YBF282) raise the question of the circulation of a single recombinant with a serotype profile of HIV-1/M or/O mono-infection.

We searched for *vpr* recombinants, because we considered this gene as a potential hot-spot of recombination; indeed, three of four MO recombinant forms previously reported exhibited a *vpr* breakpoint. This and our data show that this region could be essential for MO recombination, compared to intra-group HIV-1/M recombination. Among the HIV-1/M CRFs, reported in the Los Alamos Database, only 12 show a recombinant breakpoint in *vpr*. The only HIV-2 circulating recombinant form has no breakpoint in the *vpr* gene. But, we also found a *vpr* recombinant, because we focused on this gene; the description of two recombinant forms with a breakpoint outside *vpr,* in *vpu* here (as well as at the end of *env*) and in Integrase in a previous report [12], highlights the fact that some recombinants do not harbor such a breakpoint, and that the situation could be more complex as for HIV-1/M CRFs. Thus, we report here four cases of dual infection without *vpr* recombinant, even though they may have recombinant viruses with another breakpoint. There is a crucial need to sequence the whole genome of these partial forms to identify other breakpoints. But full-length sequencing of multiple distinct populations present in the samples is technically difficult, even by cloning; we need to optimize such tools or use next generation sequencing technology, more adapted to discriminating between numerous populations. Lastly, we analyzed RNA or DNA forms according to the material available and at a unique sampling point for each patient, but dual infections and recombination are dynamic processes, combining co-infection or superinfection, emergence and selection of recombinants, and potential disappearance of one or both parental strains. To better understand these dynamics and the emergence of more adapted forms, we need to analyze sequential samples, and both DNA and RNA to distinguish between archived and replicative forms.

In conclusion, our combined sero-molecular strategy allowed us to identify new inter-group HIV-1/MO recombinant forms, with characterization of two completely new genomes, associated or not with dual infections. This work highlights the dynamic co-evolution of these two groups of strains in Cameroon; a new study taking into account our limitations is in process to better estimate the prevalence and genetic pattern of the [MO]/[OM] recombinants.
